# Lipopolysaccharide Increases Immune Activation and Alters T Cell Homeostasis in SHIV_B'WHU_ Chronically Infected Chinese Rhesus Macaque

**DOI:** 10.1155/2015/202738

**Published:** 2015-12-02

**Authors:** Gao-Hong Zhang, Run-Dong Wu, Hong-Yi Zheng, Xiao-Liang Zhang, Ming-Xu Zhang, Ren-Rong Tian, Guang-Ming Liu, Wei Pang, Yong-Tang Zheng

**Affiliations:** ^1^Key Laboratory of Animal Models and Human Disease Mechanisms of the Chinese Academy of Sciences & Yunnan Province, Kunming Institute of Zoology, Chinese Academy of Sciences, Kunming, Yunnan 650223, China; ^2^College of Pharmacy and Chemistry, Dali University, Dali, Yunnan 671000, China; ^3^School of Life Sciences, University of Science and Technology of China, Hefei, Anhui 230026, China; ^4^University of Chinese Academy of Sciences, Beijing 100039, China

## Abstract

Immune activation plays a significant role in the disease progression of HIV. Microbial products, especially bacterial lipopolysaccharide (LPS), contribute to immune activation. Increasing evidence indicates that T lymphocyte homeostasis disruptions are associated with immune activation. However, the mechanism by which LPS affects disruption of immune response is still not fully understood. Chronically SHIV_B'WHU_-infected Chinese rhesus macaques received 50 *μ*g/kg body weight LPS in this study. LPS administration affected the virus/host equilibrium by elevating the levels of viral replication and activating T lymphocytes. LPS induced upregulation of CD8^+^ naïve T cells and downregulated the number of CD4^+^ and CD8^+^ T effector memory cells. The downregulated effector memory cells are associated with a lower frequency of monofunctional and polyfunctional cells, and an upregulated programmed cell death-1 (PD-1) expression on CD4^+^ and CD8^+^ T cells was observed in monkeys after LPS stimulation. Our data provide new insights into the function of LPS in the immune activation in SHIV/HIV infection.

## 1. Introduction

Chronic immune activation and inflammatory cytokine production are the hallmarks of HIV infection [1, 2]. It is widely accepted that immune activation levels accurately predict HIV disease progression to AIDS [3–5]. The mechanisms leading to immune activation are not yet fully clear but microbial translocation plays an important role in this process. Early loss of gut mucosal integrity results in the translocation of microbes and microbial products such as LPS into the systemic circulation [6, 7].

LPS was reported to contribute to HIV infection-related systemic immune activation. Shan and Siliciano demonstrated that blocking the translocation of intestinal bacterial LPS into the circulation dramatically reduced T cell activation and proliferation, production of proinflammatory cytokines, and plasma SIV RNA levels [8]. Administration of LPS to chronically SIVagm-infected African green monkeys triggered immune activation and viral replication and depletion of intestinal CD4^+^ T cells [9].

T cells are critical in controlling both cellular and humoral immune responses that can effectively suppress virus replication. Accumulated evidence has demonstrated that persistent immune activation results in exhaustion and dysfunction of T and B cells [10, 11]. During HIV chronic infection, sustained activation of CD4^+^ and CD8^+^ T cells is associated with depletion of CD4^+^ T cells and increased risk of disease progression to AIDS [12, 13]. T cell activation makes these cells more susceptible to HIV infection, thus creating more targets for viral replication. Few studies have addressed the dynamics and function of T cells after LPS administration. Tincati et al. investigated the role of LPS* in vitro* on T cell activation in HIV-infected patients [14].

SIV/SHIV-infected Chinese-origin rhesus macaques (Ch-RMs) serve as an important animal model for investigating the pathology of HIV [15, 16]. Over the past few years, we have successfully developed the acute and chronic macaque models of SIV infection [17, 18]. Although administration of LPS to chronic SIVmac239-infected Ch-RMs induced a transient increase of plasma SIV RNA and immune activation [19], it is still unclear whether LPS plays a role in T cell homeostasis and function during SIV/HIV infection.

Understanding the complex interplay between T cell homeostasis and LPS in experimentally SHIV-infected Ch-RMs may provide important insights into the mechanisms of microbial translocation in disease progression. In this study, we examined the dynamics and function of T cells after LPS administration in SHIV_B'WHU_ chronically infected Ch-RMs.

## 2. Materials and Methods

### 2.1. Ethics Statement

The study was carried out in accordance with the regulations of the American Association for Assessment and Accreditation of Laboratory Animal Care (AAALAC) at the Kunming Primate Research Center, Kunming Institute of Zoology, CAS. All animal experiments were performed according to the guidelines approved by the Ethics Committee of Kunming Institute of Zoology (Approval number SYDW20080125001). The animals were housed at the Animal Biosafety Level-3 (ABSL-3) laboratory of the Kunming Institute of Zoology, which were monitored daily via a telemonitoring system. The room temperature range was 20–28°C, with a relative humidity of 35–60% and a 12 hrs light-dark cycle. The animals were housed in stainless steel cages (800 mm wide, 1000 mm deep, and 1000 mm high) and fed with a standard commercial monkey diet as well as fresh fruits, vegetables, and nuts. Animals had free access to food and water* ad libitum*. Animals' conditions like overall health status, behavior, eating habits, and stool were daily observed by the veterinarian. No adverse events were seen during the study, and no animal was sacrificed. LPS injection and blood collections were performed under anesthesia with an intramuscular injection of ketamine hydrochloride (10 mg/kg body weight), and standard aseptic procedures were adopted to ensure minimum animal suffering.

### 2.2. Inoculation of Chinese Macaques with SHIV_B'WHU_

Three adult male Ch-RMs (*Macaca mulatta*) (7.7 ± 1.2 kg), aged 5–8 years, were included in our study. One Chinese macaque (P4#96065) was inoculated intravenously with 1 mL of SHIV_B'WHU_-positive plasma collected from the P3#1032 macaque [20] at 2 weeks after inoculation, while the other two monkeys (P5#04045 and P5#04091) were challenged with 1000 TCID50 SHIV_B'WHU_. SHIV_B'WHU_ viral stocks were obtained by standard peripheral blood mononuclear cell (PBMC) coculture techniques. Briefly, 1 × 10^7^ PBMCs from healthy Ch-RMs were obtained by the Ficoll-Hypaque density gradient technique, activated for 48 hrs with 5 *μ*g/mL of phytohemagglutinin A, and then mixed with 5 × 10^6^ PBMCs from P4#96065 in 2 mL of medium. After 3 hrs, the cells were washed with medium and resuspended at 2 × 10^6^ cells/mL. When the coculture was identified as positive by SIV p27 antigen enzyme-linked immunosorbent assay (ELISA), supernatants and cells were stored. The TCID50 of SHIV_B'WHU_ viral stock was titrated on TZM-bl cells.

### 2.3. LPS Treatment

The animals were given an intravenous dose of 50 *μ*g/kg body weight LPS from* Escherichia coli* 026:B6 (Sigma, MO, USA, Cat. number L2654). Animals were treated twice with LPS at 14-day intervals. All animals were aviremic at the time of LPS administration. Viral quantification and immunophenotype analysis were performed on the day before the beginning of treatment to determine the baseline level.

### 2.4. Antibodies

The following monoclonal antibodies (mAbs) that cross-reacted with rhesus macaque were obtained from BD Pharmingen (BD Biosciences, CA, USA): anti-CD3-PE/-APC-Cy7 (clone SP34-2), anti-CD4-FITC/-PerCP-Cy5.5 (clone L200), anti-CD8*α*-PE-Cy7 (clone RPA-T8), anti-CD20-PerCP-Cy5.5 (clone 2H7), anti-CD14-APC (clone M5E2), anti-HLA-DR-APC (clone L243), anti-CD28-APC (clone CD28.2), anti-CD95-FITC (clone DX2), anti-Ki67-PE (clone B56), and anti-IL-4-PerCP-Cy5.5 (clone 8D4-8). Anti-CD38-FITC (clone AT-1) mAb was obtained from STEMCELL. Anti-PD-1-PE (eBioJ105) was obtained from eBioscience (CA, USA). Anti-IL-2-FITC (clone MQ1-17H12), anti-IFN-*γ*-PE (clone 4S.B3), and anti-TNF-*α*-APC (clone Mab11) mAbs were all obtained from BioLegend (CA, USA).

### 2.5. Absolute Quantification of Major Leukocyte Subpopulations

Direct cell surface staining for whole blood and absolute number analysis were performed according to standard procedures and appropriate concentrations in this study. Briefly, 50 *μ*L of whole blood was added in a TruCount tube (BD Biosciences) and incubated with fluorochrome-conjugated antibodies of CD3, CD4, CD8*α*, CD20, and CD14 for 30 min on ice. Erythrocytes were lysed with FACS lysing solution (BD Biosciences), and the samples were analyzed with a BD FACSVerse cytometer. The absolute numbers of T cells (CD3^+^CD20^−^), CD4^+^ T cells (CD4^+^CD8a^−^CD3^+^), CD8^+^ T cells (CD4^−^CD8a^+^CD3^+^), B cells (CD3^−^CD20^+^), and monocytes (CD3^−^CD20^−^CD14^+^) were calculated using the following formula: cell concentration = (events in gated region × total number of TruCount beads)/(number of acquired beads × sample volume).

### 2.6. Immunophenotyping of T Lymphocytes

For determination of activated (CD38^+^HLA-DR^+^ or PD-1^+^) T cells, naïve T cells (CD28^+^CD95^−^), central memory (TCM, CD28^+^CD95^+^), and effector memory T cells (TEM, CD28^−^CD95^+^), 100 *μ*L of fresh whole blood was lysed with Lysing Buffer (BD Biosciences) for 10 min at room temperature, followed by washing and resuspension with Dulbecco's phosphate-buffered saline (DPBS) with 2% newborn calf serum and 0.09% sodium azide (staining buffer). The suspending leukocytes were then stained with the relevant directly conjugated mAbs for 30 min on ice and fixed using PBS containing 4% paraformaldehyde.

### 2.7. Ki67 Staining and Intracellular Cytokine Staining (ICS)

For Ki67 staining, 1 × 10^6^ thawed PBMCs were stained with relevant surface marker mAbs, then fixed and permeabilized using Cytofix/Cytoperm solution (BD Biosciences), washed in Perm/Wash Buffer (BD Biosciences), and incubated with anti-Ki67 PE mAb in Perm/Wash Buffer. After washing once, cells were fixed using PBS containing 2% paraformaldehyde and then resuspended in staining buffer. For ICS, after stimulation with phorbol 12-myristate 13-acetate (PMA, 50 ng/mL) and ionomycin (1 *μ*M) for 6 hrs, PBMCs were stained with anti-CD3 and anti-CD8 mAbs on cell surface and anti-IL-2, anti-IL-4, anti-IFN-*γ*, and anti-TNF-*α* mAbs intracellularly. Analysis of the acquired data was performed using FlowJo software (version 7.6.1; TreeStar).

### 2.8. Detection of Plasma Soluble CD14 (sCD14) by ELISA

To verify that the Ch-RMs treated with LPS generated an effective response, we tested sequential plasma samples from all treated monkeys. Plasma sCD14 levels were measured using a commercially available sCD14 ELISA (R&D Systems, USA). Plasma was diluted to 1/200 and assays were performed in duplicate according to the manufacturer's protocol.

### 2.9. Absolute Quantitation of SHIV_B'WHU_ Viral Loads in Plasma

Plasma samples were analyzed for SHIV vRNA using a real-time quantitative RT-PCR assay (TOYOBO, Japan) that provides a threshold sensitivity of 100 copies/mL as previously described [21]. Briefly, vRNA was extracted using the High Pure Viral RNA Kit (Roche) according to the manufacturer's instructions. RT-qPCR assay using the RNA-direct real-time PCR master mix was performed on a 7500 Fast Real-Time PCR System (Applied Biosystems, USA).

## 3. Results

### 3.1. Efficient Infection of R5 SHIV_B'WHU_ in Ch-RMs

**Figure 1:**
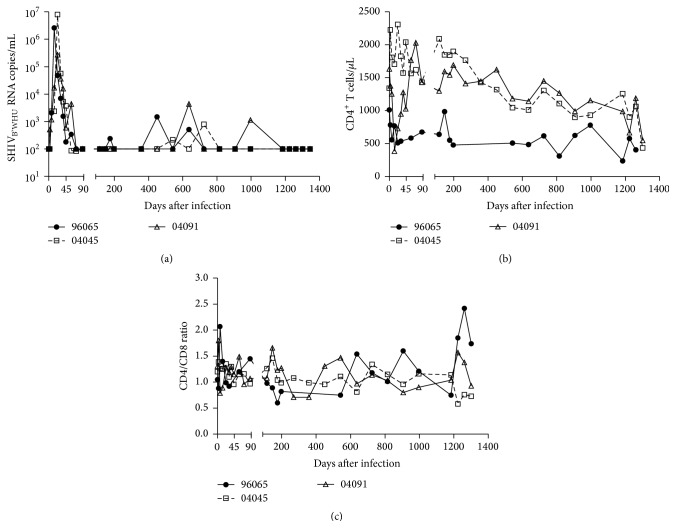
Dynamics of virologic and immunologic parameters of SHIV_B'WHU_ infection in Ch-RMs. (a) Viral replication. The dashed line indicates the limit of detection at 100 copies/mL plasma. (b) CD4^+^ T cells. (c) Ratio of CD4^+^ to CD8^+^ T cells.

SHIV_B'WHU_ was generated from SHIV_SF33_ by replacing its counterparts with tat/rev/vpu/env genes derived from a CCR5-tropic, subtype B' strain of a Chinese HIV-positive patient [20]. To determine transmissibility and pathogenicity of R5 SHIV_B'WHU_ in Ch-RMs, we inoculated three males intravenously with plasma from SHIV_B'WHU_-infected Ch-RM (#96065) or SHIV_B'WHU_ virus stock (#04045 and #04091). All inoculated animals became infected. Plasma viremia peaked at 3 weeks after infection to 6-7 log_10_⁡ RNA copies/mL in animals #04045 and #04091, and animal #96065 peaked at 2 weeks after infection (Figure 1(a)). All three animals' viral load reached undetectable levels (<100 RNA copies/mL plasma) after 3 months after infection, with partial rebound to <4 log_10_ RNA copies/mL plasma. The infected animals #04045 and #04091 experienced a gradual decline in CD4^+^ T lymphocytes despite low viral load (<10^4^ RNA copies/mL plasma). Absolute number of CD4^+^ T cells decreased by approximately 67% in the two animals (the mean values of CD4^+^ T cells decreased from 1487 cells/*μ*L at baseline to 490 cells/*μ*L at day 1300) (Figure 1(b)). The exception was the infected rhesus #96065; its peripheral CD4^+^ T cell counts remained relatively constant during infection. All animals had a persistently low CD4^+^/CD8^+^ ratio during SHIV_B'WHU_ infection, and no significant differences were found after LPS administration (Figure 1(c)). We concluded that SHIV_B'WHU_ transmits efficiently in Ch-RMs, and the infection is frequently controlled.

### 3.2. LPS Increases Viral Loads and Downregulates CD20^+^ B Cells in SHIV_B'WHU_-Infected Ch-RMs

**Figure 2:**
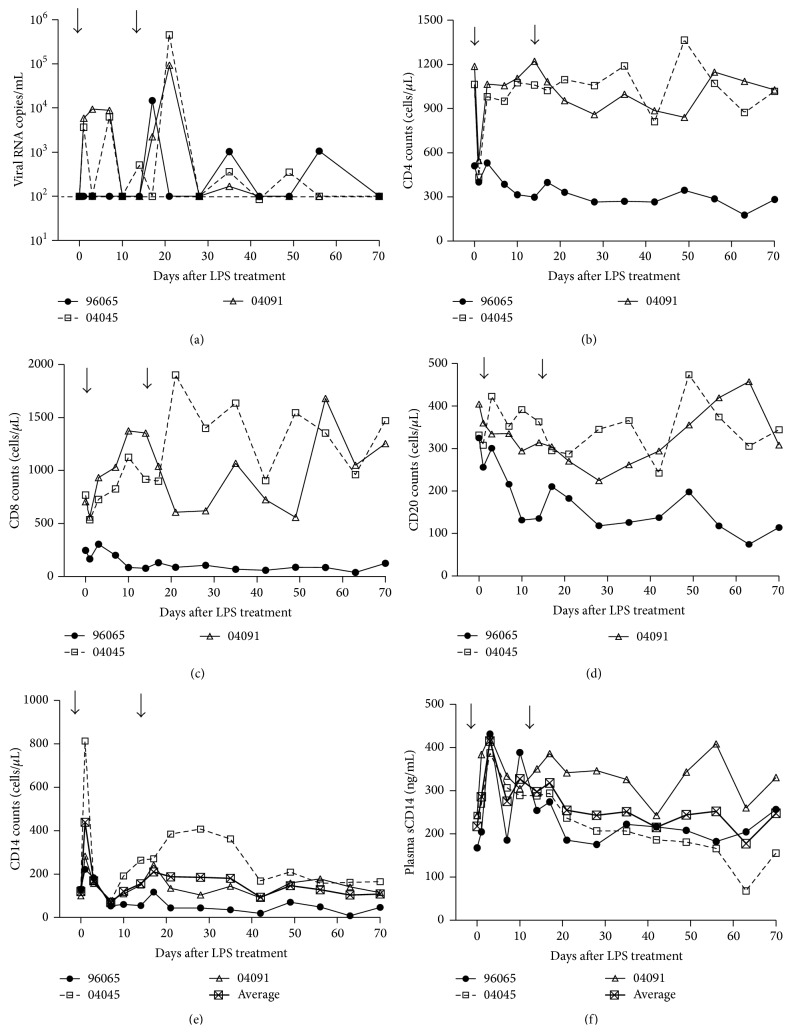
LPS administration in chronically SHIV_B'WHU_-infected Ch-RMs. LPS induces a transient increase in the VL. The dashed line indicates the limit of detection at 100 copies/mL plasma (a), a transient decrease in the absolute number of CD4^+^ T cells (b), an increase in CD8^+^ T cells (c), a decrease in CD20^+^ B cells (d), an increase in levels of CD14^+^ cells (e), and an increase in plasma sCD14 (f). Ch-RMs were intravenously injected twice with LPS at 50 *μ*g/kg body weight at day 0 and day 14 (arrows).

Macaques at steady phase of chronic infection (4 years after infection for #04045 and #04091, 5.5 years after infection for #96065) received LPS intravenously twice at 14-day intervals at a dose of 50 *μ*g/kg body weight. Our results showed that LPS at low doses can be given safely, without systemic side effects. As expected, LPS administration resulted in a transient but very rapid increase in plasma viral load (VL) (Figure 2(a)), and increased VL was detected at day 1 or day 3 after LPS treatment. The absolute numbers of CD4^+^ T cell decreased in all monkeys at day 1 from a mean of 920 to 460 after LPS administration and returned to baseline rapidly at day 3 (Figure 2(b)). In addition, the effect of LPS treatment on CD8^+^ T cell counts was even more pronounced with 1.5- and 2-fold increases from baseline in the two animals #04045 and #04091 treated at day 10 (Figure 2(c)).

Recent studies showed loss of memory B cells in the majority of acute and chronic phases of SIV/HIV infection [22, 23]. Microbial translocation might play a role in HIV-associated B cell perturbations [24]. Interestingly, flow cytometric assessment demonstrated a downregulation of CD20^+^ B cells in the peripheral blood at most time points after LPS administration in our study. The dynamic B cell population in SHIV-infected macaques downregulates rapidly after LPS stimulation. Further studies are needed to completely elucidate the mechanism of CD20^+^ B cell regulation (Figure 2(d)).

Plasma levels of sCD14 indicate the degree of bacterial translocation and predict disease progression in HIV-1 patients [25, 26]. In agreement with previous studies, plasma sCD14 levels were elevated in the SHIV_B'WHU_-infected monkeys after LPS administration. As shown in Figure 2(e), the average CD14^+^ cell levels increased from a baseline value of 119 cells/*μ*L to 439 cells/*μ*L at day 1 of LPS administration (Figure 2(e)), and the average plasma sCD14 levels also increased from a baseline value of 218 ng/mL to 415 ng/mL at day 3 (Figure 2(f)).

### 3.3. LPS Transiently Induces Proliferation and Activation of T Cells

**Figure 3:**
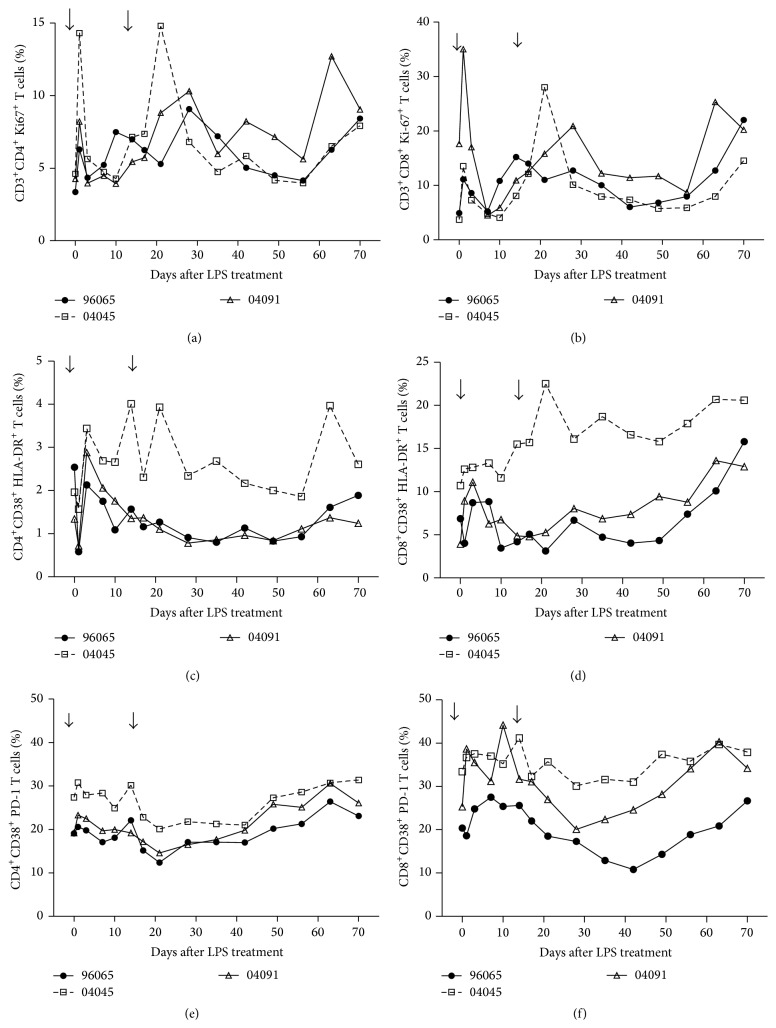
Impact of LPS administration on T cells in chronically SHIV_B'WHU_-infected Ch-RMs. Increases of percentages of Ki-67^+^/CD4^+^ T cells (a) and Ki-67^+^/CD8^+^ T cells (b) were observed after administration of LPS. Increases of percentages of CD38^+^HLA-DR^+^CD4^+^ (c) and CD38^+^HLA-DR^+^CD8^+^ T cells (d) were observed after administration of LPS. LPS did not induce remarkable changes of PD-1 on activated CD4^+^ (e) and CD8^+^ T cells (f).

CD8^+^ and CD4^+^ T cells play an important role in controlling HIV-1 replication and disease progression. Next we evaluated the proportion of proliferating CD4^+^ and CD8^+^ T cells as assessed by the percentages of T cells expressing nuclear Ki67 antigen. Our results showed a transient and nonsignificant increase in Ki67 expression in peripheral CD4^+^ and CD8^+^ T cells (Figures 3(a) and 3(b)). In animal #04045, we found an increase of Ki67-expressing CD4^+^ T cells from 4.6% before treatment to 14.3% at day 1 (3.1-fold increase) after treatment. In animals #96065 and 04091, the Ki67-expressing CD4^+^ T cell population rose from 3.4% and 4.3% before treatment to 6.3% and 8.2% at day 1 (1.85- and 1.9-fold increase, resp.). Similarly, LPS treatment transiently increased Ki67-expressing CD8^+^ T cells at day 1 after treatment by 2- to 3.6-fold.

We then assessed the activation status of CD4^+^ and CD8^+^ T cell subsets before and after LPS treatment. LPS stimulation resulted in upregulation of CD38 and HLA-DR on CD8^+^ T cells (Figure 3(d)). The average percentage of CD38^+^HLA-DR^+^ expressing CD8^+^ T cells increased from a baseline value of 7% to 11% at day 3. LPS showed only a weak impact on the frequency of CD38^+^HLA-DR^+^ expressing CD4^+^ T cells after the second LPS injection compared to that observed in CD4^+^ T cells after the first injection on monkeys #96065 and #04091. Monkey #04045 showed a significant increase of the percentage of CD4 and CD8 cells with the second LPS injection (Figure 3(c)).

### 3.4. LPS Upregulates PD-1 Expression on CD4^+^ and CD8^+^ T Cells

HIV-1 infection is associated with functional impairment of HIV-1-specific CD8^+^ and CD4^+^ T cells. Elevated programmed death-1 (PD-1) expression on the surface of CD4^+^ and CD8^+^ T cells in HIV-1 infection is associated with T cell exhaustion [27]. To address whether T cells of the LPS-treated animals were functionally impaired, we examined the expression of PD-1 on T cells before and after LPS injection. PD-1 expression was increased following the first stimulation with LPS on both CD4^+^ and CD8^+^ T populations (Figures 3(e) and 3(f)). We have therefore addressed the hypothesis that LPS modulates PD1 expression on T cells. PD-1 expression on T cells might be driven by viral replication and associated with T cell dysfunction.

### 3.5. LPS Has a Differential Effect on CD4^+^ and CD8^+^ T Cell Subpopulations

As we observed a transient activation of CD4^+^ and CD8^+^ T cells after treatment with LPS, we next examined whether T cell subpopulations could be changed upon LPS stimulation. Thus, we evaluated the effects of LPS on T cell subpopulation distribution defined by the expression of CD95 and CD28. CD95^−^CD28^+^ were identified as naïve T cells, CD95^−^CD28^−^ were identified as TEM cells, and CD95^+^CD28^+^ were identified as TCM cells.

**Figure 4:**
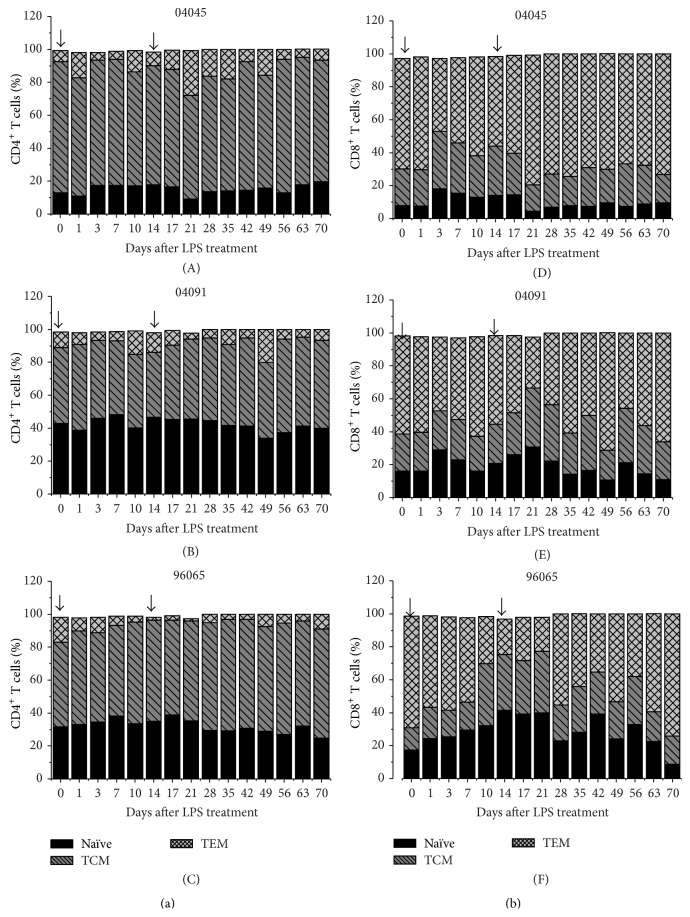
Frequency of naïve and memory CD4^+^ or CD8^+^ T cells in LPS-treated Ch-RMs. CD3^+^CD4^+^ (a) and CD3^+^CD8^+^ (b) T cells were assessed for CD28 and CD95 expression to identify naïve (CD28^+^CD95^−^), central memory (TCM; CD28^+^CD95^+^), and effector memory (TEM; CD28^−^CD95^+^) cell subsets. The percentages of naïve CD8^+^ T cells increased after LPS administration, and effector memory CD8^+^ T cells decreased compared to baseline values. Baseline values were taken prior to LPS administration.

We found that the relative frequencies of T cell subpopulations in blood vary in the CD4^+^ and CD8^+^ cells. TCM was predominant in CD4 and TEM was predominant in CD8. Interestingly, our data revealed that LPS treatment had a differential effect on CD4^+^ and CD8^+^ T cell subset distribution. As seen in Figure 4(b), LPS provoked a rapid increase in the frequency of CD8^+^ naïve T cells compared with pretreatment values. This frequency then returned to basal levels at day 14 after LPS treatment. Conversely, no differences in CD4^+^ naïve T cells were observed after LPS treatment (Figure 4(a)). LPS induced a slight reducing effect on CD4^+^ TEM cell subsets, whereas treatment with LPS led to a more selective inhibitory effect on CD8^+^ TEM. Percentages of CD8^+^ TEM cells decreased by 17% and 58% in treated animals.

### 3.6. Th1 Cytokine Responses Are Perturbed after LPS Stimulation

Cytokines play a key role in controlling HIV-1 replication and T cell regulation. HIV-1 infection induced a progressive loss of T cell function including lowered capacity to produce cytokines. Understanding the cytokine networks in CD4^+^ and CD8^+^ T cells during SHIV infection is crucial for the control of viral replication.

**Figure 5:**
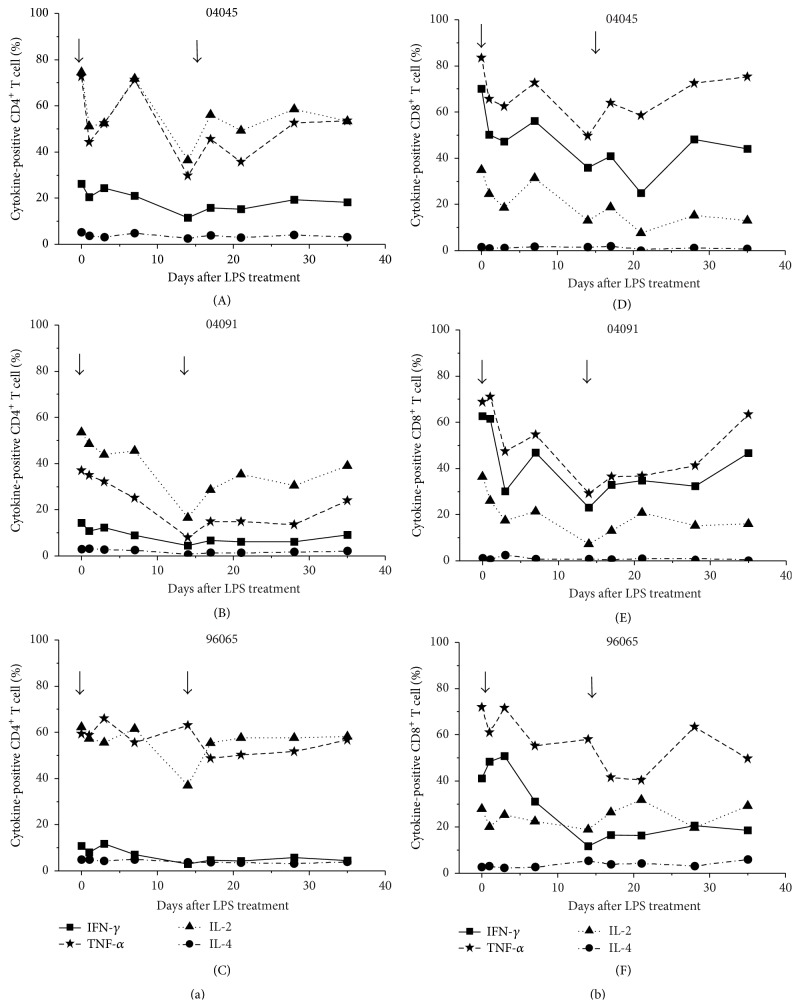
Cytokine profile of CD4^+^ and CD8^+^ T cells in response to PMA and ionomycin stimuli. PBMCs were isolated and stimulated by PMA and ionomycin as described in the Materials and Methods and analyzed by flow cytometry for intracellular production of IFN-*γ*, TNF-*α*, IL-2, and IL-4. Proportion of cytokine-producing CD4^+^ T cells in response to stimulus (a); proportion of cytokine-producing CD8^+^ T cells in response to stimulus (b).

Th1 (IFN-*γ*, IL-2, and TNF) and Th2 (IL-4) cytokines expressing CD4^+^ and CD8^+^ T cells were assayed after LPS injection. IL-4^+^ T cells were present at low frequencies over the course of LPS administration, and their frequency did not change in response to stimulation with LPS. CD4^+^ T cell responses after LPS injection included decreased frequencies of IL-2^+^CD4^+^, TNF-*α* ^+^ CD4^+^, and TNF-*α* ^+^CD4^+^ T cells in monkeys #04045 and #04091, but not #96065, relative to the pre-LPS period (Figure 5(a)). The treated animals experienced a transient pulse of decrease in the frequency of IFN-*γ* ^−^, IL-2^−^, and TNF-*α* ^−^ expressing CD8^+^ T cells after LPS injection in all treated monkeys (Figure 5(b)).

**Figure 6:**
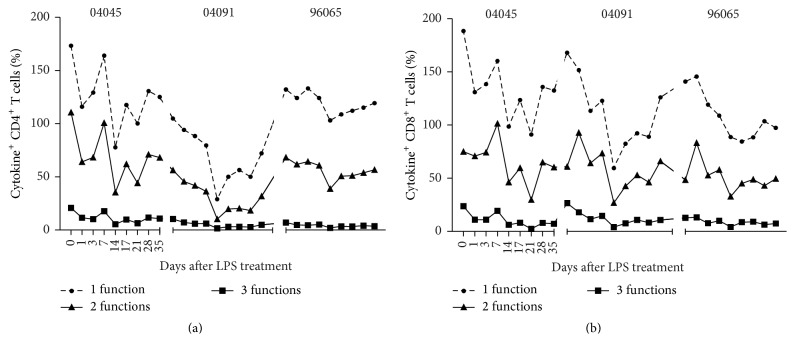
Frequency of cytokine-positive T cells in LPS-treated Ch-RMs. Cytokine-positive T cells were grouped into three categories: IFN-*γ* ^+^TNF-*α* ^+^IL-2^+^ 3-function cells (filled squares), 2-function cells (filled triangles) expressing two cytokines, and 1-function cells expressing only IFN-*γ*, TNF-*α*, or IL-2 (filled circles).

Multifunctional T cells are the potential correlates of protection against HIV. We tested the multifunctionality of T cell responses in SHIV_B'WHU_-infected monkeys before and after treatment with LPS. Most cytokine-expressing cells were positive for only a single cytokine (Figures 6(a) and 6(b)). There was a drop in single-positive cells concurrent with decreased frequencies of multifunctional cells, suggesting that the function of T cells is destroyed. The animals had fewer Th1-polarized T cells after LPS treatment than baseline levels. These data suggest that, in addition to activating T cells, LPS influences their functional phenotypes. These findings suggest that changes in T cell function during LPS stimulation become important factors for immune activation and viral replication inhibition.

### 3.7. Changes in Hematological Parameters of SHIV Monkeys after LPS Treatment

**Figure 7:**
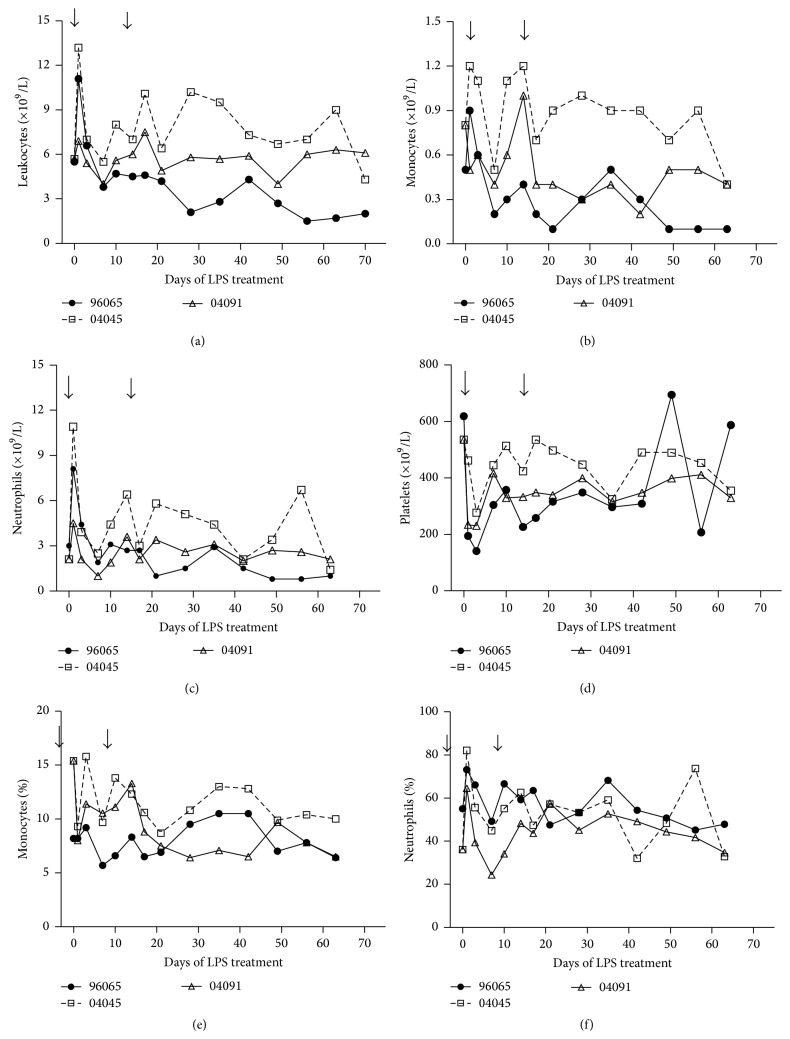
Hematological changes associated with LPS administration in Ch-RMs. Levels of leukocytes (a) were increased in LPS-treated Ch-RMs with concomitant increases in monocytes (b) and neutrophils (c). Platelet counts (d) were decreased after LPS stimulation. The percentages of monocytes (e) and neutrophils (f) were increased in blood.

The basic complete blood count (CBC) is an important parameter for HIV infection. To identify hematological changes that are associated with LPS treatment, we examined longitudinal hematology data during LPS treatment. Most hematological parameters remained unchanged during LPS treatment (data not show), while the number of leukocytes, monocytes, and neutrophils increased rapidly at day 1 in response to LPS treatment (Figures 7(a), 7(b), and 7(c)).

The leukocytes are the first line of immune defense against infection and respond rapidly to LPS. Elevated levels of leukocytes can be the result of inflammatory response and immune activation. A temporary drop in the circulating platelet counts were observed at day 1 after LPS treatment. In animal #96065, the platelet count decreased by 77% at day 3. In the other two animals #04045 and 04091, the platelet count decreased by 48% and 57% at day 3, respectively (Figure 7(d)). Our findings are consistent with previous reports showing a decrease in circulating platelets during SIV/HIV infection [28, 29]. We speculate that the decrease of platelet counts may be caused by increased activation and subsequent sequestration of platelets in platelet-monocyte aggregates as reported by Metcalf Pate et al. [30].

## 4. Discussion

Persistent immune activation is a hallmark of progressive HIV infection. The level of immune activation is more closely associated with disease progression compared to plasma VL [31]. The breakdown of intestinal mucosal integrity in HIV infection leads to translocation of bacterial products (LPS, DNA) into systemic circulation. Plasma levels of LPS are closely associated with the intestinal permeability degree. Plasma LPS level may be a useful marker of gut injury in chronically HIV-infected patients. LPS levels are usually measured to determine the degree of microbial translocation in HIV/SIV infection [32]. Accumulative evidence indicates that microbial translocation (MT) promotes systemic immune activation in chronic HIV infection [1]. As previously reported, the translocation of LPS contributes to the systemic immune activation in HIV-1 infection [33], and microbial translocation (especially LPS) has been associated with HIV disease progression [34, 35].

In the current study, we demonstrated that LPS administration affected the virus/host equilibrium. LPS treatment induced a rapid, transient increase in activated T cells. The administration of LPS into peripheral blood causes T cell activation, activated CD4^+^ T cells are highly susceptible to infection by HIV-1, and the increased viral replication may further exacerbate the immune activation. In our study, increased levels of LPS were associated with increased T cell activation and proliferation and increased production of HIV in LPS-treated animals, suggesting that LPS administration directly causes viral associated immune activation. The results are in agreement with a recent study showing augmentation of viral replication and immune activation after LPS injection in SIVmac239-infected Ch-RMs [19] and with data reporting increased levels of VL were triggered by the LPS in chronically SIVagm-infected African green monkeys [9]. The levels of B cells were downregulated after LPS administration in SHIV-infected monkeys in our study, and a few days later the cell number increased again, which is consistent with a previous report showing that LPS and HIV synergistically induce memory B cell apoptosis [36].

We examined the* in vivo* activation and proliferation of T cells, the relative expression of PD-1 and cytokine, and the T cell subset distribution in chronically SHIV-infected RMs during LPS administration. Treatment with LPS has a different effect on CD4^+^ and CD8^+^ T cell subset repartition (Figure 4). As shown in Figure 4 CD95^−^CD28^−^CD8^+^ cells were named TEM, and the proportion of these CD8^+^ TEM cells was decreased in treated animals, whereas the naïve CD95^−^CD28^+^CD8^+^ T cell population was increased as compared with levels in pretreated animals (Figure 4). The CD4^+^ TEM cell subpopulation was decreased in treated animals, whereas the naïve and central memory phenotype of CD4^+^ T cells did not differ during the LPS injection (Figure 4). These results are consistent with the findings of Catalfamo et al., who reported the forces that lead to immune dysfunction difference for CD4^+^ and CD8^+^ T cells [37]. The CD95^−^CD28^−^CD8^+^ effector memory T cells provide an immediate effector response to viral infection and exhibit lytic activity. They have the potential to rapidly produce cytokines and eliminate infected cells [38]. The decreased numbers of CD95^−^CD28^−^ effector memory CD8^+^ T cells in peripheral blood may reflect increased apoptosis of these cells or enhanced migration of these cells to sites of inflammation, which may play a role in the pathogenesis of LPS.

Functional impairment of CD8^+^ and CD4^+^ T cells eventually results in a progressive failure of the immune system to control HIV-1. Increasing data have shown that PD-1 expression on HIV-specific T cells is generally associated with T cell exhaustion during HIV-1 infection [39–41]. In SIV-infected rhesus macaques, high PD-1 expression is associated with SIV-specific T cell dysfunction during acute and chronic infection. Blockade of the PD-L1/PD-1 pathway* in vivo* in chronic SIV-infected monkeys reduces immune activation and restores the function of cellular and humoral immune responses [42, 43]. PD-1 expression is associated with cytokine production as well as T cell expansion [44]. IFN-*γ* and tumor necrosis factor-*α* (TNF-*α*) are the most common cytokines associated with HIV protection. IL-2 acts in conjunction with IFN-*γ* and TNF-*α* by promoting T cell survival and proliferation. The cytokine-releasing capacity is also an important function of T cells against HIV infection. A great deal of evidence indicates that multifunctional CD8^+^ T cells are essential for controlling HIV replication and disease progression. Elite controllers have CD8^+^ T cells that are more polyfunctional than HIV-infected progressors [45]. In HIV-infected individuals, the presence of polyfunctional T cells has been associated with superior control of viral infection [46, 47].

In this study, CD4^+^ and CD8^+^ T cells showed defects in function after LPS administration in SHIV_B'WHU_-infected monkeys. First, reduced cytokine expression of CD4^+^ and CD8^+^ T cells after LPS treatment was detected in this model. Secondly, the expression levels of PD-1 on CD4^+^ and CD8^+^ T cells were increased in posttreatment animals compared to those in pretreatment animals (Figure 3(f)). We showed here that the upregulation of PD-1 in T cells after LPS administration is associated with alterations in the distribution of T cell subpopulations and with impaired expression of cytokines. We reconfirmed the findings that PD-1 can be used as a marker for aberrant distribution of T cell subpopulations in HIV-1 infection [48]. We speculated here that immune activation and increased HIV RNA induced by LPS treatment may contribute to the loss of functional T cell responses.

A potential limitation of the present study is the smaller animal size. However, it is important to consider that the experiment was performed in a controlled system. At the time of LPS injection, SHIV_B'WHU_-infected animals had normal CD4^+^ T cell counts and showed no clinical symptoms. The remarkable stability of viral loads in SHIV_B'WHU_-infected Ch-RMs model (low levels of chronic viremia maintained for decades) allows accurate detection of discrete alterations in immune activation and proliferation during LPS treatment.

In summary, our data provided a direct relationship between LPS and immune activation. LPS can directly stimulate immune activation, making more target cells available for viral exploitation. Increased viral replication in target cells may in turn exacerbate these changes and result in an altered T cell homeostasis during chronic HIV infection. Our data thus advocate further functional studies to gain deeper insight into the regulation of immune response by microbial products in course of SIV/HIV disease.
